# Randomized controlled trial of a positive affect intervention to reduce HIV viral load among sexual minority men who use methamphetamine

**DOI:** 10.1002/jia2.25436

**Published:** 2019-12-20

**Authors:** Adam W Carrico, Torsten B Neilands, Samantha E Dilworth, Jennifer L Evans, Walter Gόmez, Jennifer P Jain, Monica Gandhi, Steven Shoptaw, Keith J Horvath, Lara Coffin, Michael V Discepola, Rick Andrews, William J Woods, Daniel J Feaster, Judith T Moskowitz

**Affiliations:** ^1^ University of Miami School of Medicine Miami FL USA; ^2^ San Francisco School of Medicine University of California San Francisco CA USA; ^3^ Berkeley School of Social Welfare University of California Berkeley CA USA; ^4^ San Diego School of Medicine University of California La Jolla CA USA; ^5^ Departments of Family Medicine and Psychiatry Los Angeles David Geffen School of Medicine University of California Los Angeles CA USA; ^6^ Department of Psychology San Diego State University San Diego CA USA; ^7^ San Francisco AIDS Foundation San Francisco CA USA; ^8^ Department of Medical Social Sciences Northwestern University Chicago CA USA

**Keywords:** contingency management, HIV, men who have sex with men, methamphetamine, mindfulness, positive affect

## Abstract

**Introduction:**

In the era of HIV treatment as prevention (TasP), evidence‐based interventions that optimize viral suppression among people who use stimulants such as methamphetamine are needed to improve health outcomes and reduce onward transmission risk. We tested the efficacy of positive affect intervention delivered during community‐based contingency management (CM) for reducing viral load in sexual minority men living with HIV who use methamphetamine.

**Methods:**

Conducted in San Francisco, this Phase II randomized controlled trial tested the efficacy of a positive affect intervention for boosting and extending the effectiveness of community‐based CM for stimulant abstinence to achieve more durable reductions in HIV viral load. From 2013 to 2017, 110 sexual minority men living with HIV who had biologically confirmed, recent methamphetamine use were randomized to receive a positive affect intervention (n = 55) or attention‐control condition (n = 55). All individual positive affect intervention and attention‐control sessions were delivered during three months of community‐based CM where participants received financial incentives for stimulant abstinence. The 5‐session positive affect intervention was designed to provide skills for managing stimulant withdrawal symptoms as well as sensitize individuals to natural sources of reward. The attention‐control condition consisted of neutral writing exercises and self‐report measures.

**Results:**

Men randomized to the positive affect intervention displayed significantly lower log_10_ HIV viral load at six, twelve and fifteen months compared to those in the attention‐control condition. Men in the positive affect intervention also had significantly lower risk of at least one unsuppressed HIV RNA (≥200 copies/mL) over the 15‐month follow‐up. There were concurrent, statistically significant intervention‐related increases in positive affect as well as decreases in the self‐reported frequency of stimulant use at six and twelve months.

**Conclusions:**

Delivering a positive affect intervention during community‐based CM with sexual minority men who use methamphetamine achieved durable and clinically meaningful reductions in HIV viral load that were paralleled by increases in positive affect and decreases in stimulant use. Further clinical research is needed to determine the effectiveness of integrative, behavioural interventions for optimizing the clinical and public health benefits of TasP in sexual minority men who use stimulants such as methamphetamine.

## 1 Introduction

In the era of HIV treatment as prevention (TasP), novel approaches are needed to maximize the clinical and public health benefits of anti‐retroviral therapy (ART) in high priority populations such as sexual minority men (i.e. gay, bisexual and other men who have sex with men) who use methamphetamine 1. ART to achieve viral suppression (i.e. viral load <200 copies/mL) optimizes health outcomes among people living with HIV and also dramatically reduces onward HIV transmission rates 2. However, people who use stimulants such as methamphetamine are more likely to have difficulties with ART adherence 3 and consequently experience viral suppression more slowly 4. Achieving and maintaining viral suppression is essential for better health outcomes, and cohort studies have consistently observed that more frequent stimulant users receiving ART display hastened clinical HIV progression 5, 6, 7. Because stimulant users are also more likely to engage in HIV transmission risk behaviour 8, unsuppressed viral load amplifies risk of onward HIV transmission 9.

Behavioural interventions such as cognitive‐behavioural therapy and contingency management (CM) have demonstrated moderate effectiveness, but important concerns remain about the durability of treatment gains 10, 11. CM is an evidence‐based behavioural intervention where individuals receive tangible incentives for health behaviour change such as biologically confirmed stimulant abstinence. CM is effective with methamphetamine users as a stand‐alone therapy 12, 13 and has been shown to enhance the effectiveness of substance use disorder treatment 13, 14. At the same time, there is a clear need for novel approaches to achieve more durable behaviour change after the CM incentives are discontinued. This is evident in the results of two randomized controlled trials (RCTs) with diverse samples of people living with HIV who use substances where CM interventions achieved short‐term reductions in viral load that were not maintained following the 6‐month intervention period 15, 16.

Combination interventions are needed to target fundamental neurobehavioural processes such as withdrawal and anhedonia in people living with HIV who use substances that stem from pathophysiologic alterations in the dopamine reward system as well as dysfunction in the pre‐frontal cortex 17, 18. Specifically, behavioural interventions that cultivate positive affect such as happiness or gratitude could boost and extend the effectiveness of CM by providing skills for managing stimulant withdrawal as well as sensitizing individuals to natural sources of reward 19. In fact, Revised Stress and Coping Theory proposes that positive affect serves a key adaptive role in the midst of chronic stress to reinvigorate cognitive‐behavioural coping responses 20. This is supported in part by findings that positive affect is associated with decreased stimulant use, better ART adherence and persistence, and lower HIV viral load 21, 22, 23.

There is emerging evidence for the efficacy of theory‐based positive affect interventions in various populations 24, 25, 26. Positive affect interventions generally incorporate multiple components including mindfulness‐based meditation practices designed to cultivate greater insight and awareness that are thought to be essential to experiencing positive emotions 27, 28, 29, 30, 31. In one RCT with people who were recently diagnosed with HIV, we observed that a positive affect intervention significantly improved psychological adjustment and decreased antidepressant medication use compared to an attention‐control condition 24. In that RCT, there was also a provocative trend towards intervention‐related increases in viral suppression that did not reach statistical significance.

Conducted in San Francisco, this Phase II RCT examined the efficacy of a positive affect intervention for achieving more durable reductions in viral load following community‐based CM for stimulant abstinence in sexual minority men living with HIV who use methamphetamine 32. We examined the efficacy of the positive affect intervention delivered during community‐based CM would for achieving more durable reductions in log_10_ HIV viral load over 15 months, the primary outcome. We also examined the efficacy of the positive affect intervention delivered during community‐based CM with respect to key secondary outcomes over the 15‐month follow‐up period including: any unsuppressed HIV viral load (i.e. ≥200 copies/mL), T‐helper (CD4^+^) cell count, positive affect and stimulant use.

## 2 Methods

### 2.1 Recruitment and screening

**Figure 1:**
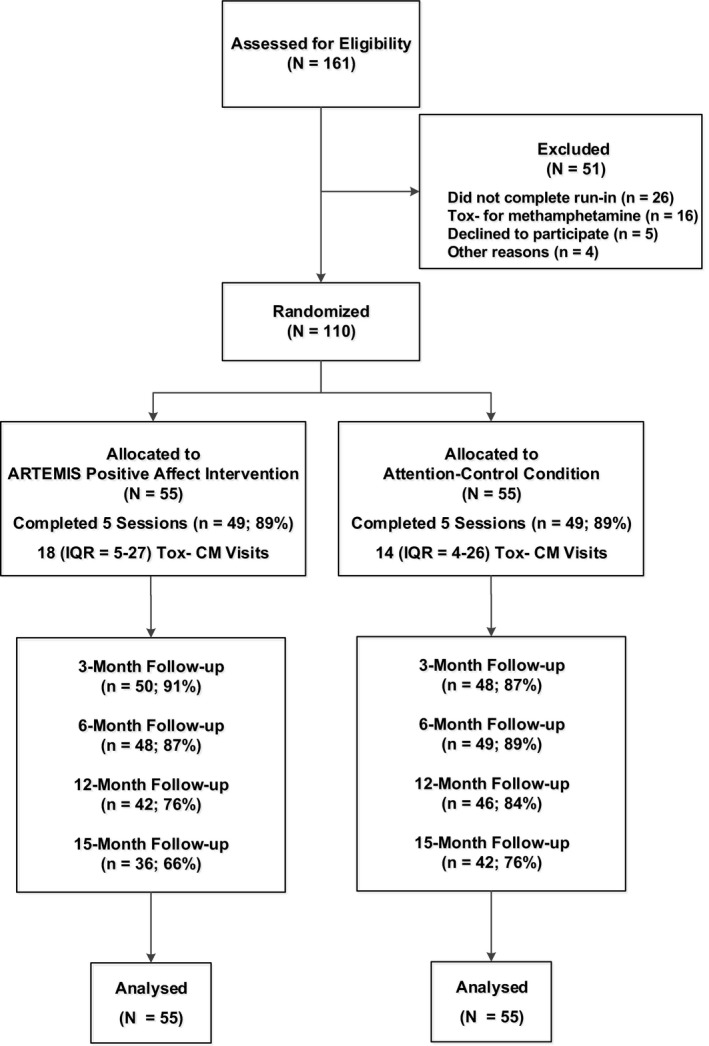
Screening, randomization and follow‐up for participants.

From 2013 to 2017, a total of 184 individuals were recruited for this RCT in the San Francisco Bay Area from a community‐based CM programme, with flyers and palm cards distributed in the community, and via an incentivized snowball sampling method where eligible participants received up to $30 for referring other eligible participants. To be eligible for this Phase II RCT, participants were required to meet the following inclusion criteria: (1) 18 years of age or older; (2) report anal sex with a man in the past 12 months; (3) speak and read English; (4) provide documentation of HIV‐positive serostatus (i.e. letter of diagnosis or being on ART medications other than tenofovir disoproxil fumarate/emtricitabine that were matched via photo identification); and (5) provide a urine or hair sample that was reactive for methamphetamine. As shown in Figure 1, of the 161 participants who completed a screening visit 16 (10%) were excluded because they did not test reactive for methamphetamine, five (3%) declined to participate and four (2%) did not meet other inclusion criteria. Participants could receive up to $400 and an iPod shuffle for completing all research‐related visits for this RCT. This included $50 for completing each of the six assessment visits and $20 for completing each of the five intervention or attention‐control sessions.

All relevant procedures were approved by the Institutional Review Board for the University of California, San Francisco with reliance agreements from the University of Miami and Northwestern University (http://www.clinicaltrials.gov identifier NCT01926184). All participants completed a signed informed consent and a certificate of confidentiality was provided by the National Institute on Drug Abuse. The University of California, Los Angeles Data Safety and Monitoring Board for Addiction Medicine held annual meetings to review participant‐related events and overall progress for this RCT. There were no adverse events or serious adverse events.

### 2.2 Run‐in period and randomization

Eligible participants completed a waiting period prior to randomization (i.e. run‐in) that entailed five separate visits: (1) a baseline assessment with a peripheral venous blood sample; (2) three CM urine screening visits (regardless of the toxicology results); and (3) a separately scheduled randomization visit where the first session of the positive affect intervention or attention‐control condition was delivered after randomization. Participants who did not complete the run‐in period were not randomized. Of the 136 participants who were eligible and consented to participate in the RCT, 110 (81%) completed the run‐in period and were randomized during the first eight weeks of CM. Randomization was accomplished using a computer generated sequence with randomly permuted block sizes of 2, 4, and 6 to guard against subversion. Only the study data manager had access to the computer‐based randomization algorithm.

### 2.3 Assessments

Participants completed a baseline assessment during the run‐in period that included self‐report measures, a urine sample for on site toxicology screening, and a peripheral venous blood sample. Participants completed follow‐up assessments at three, six, twelve and fifteen months after the beginning of CM that included computer‐based administration of self‐report measures and a urine sample for on site toxicology screening. Peripheral venous blood samples to measure HIV disease markers were collected at baseline, six, twelve and fifteen months. In the 110 participants randomized, follow‐up rates at 3 (89%), 6 (88%), 12 (80%) and 15 (71%) months were acceptable with no significant differences between the experimental conditions. Follow‐up assessments were completed in August of 2018.

### 2.4 Community‐based CM programme

This RCT was conducted in partnership with a community‐based, 3‐month CM programme for sexual minority men who use methamphetamine that is operated by the San Francisco AIDS Foundation 33. CM was delivered separately from the individual intervention or attention‐control sessions. Urine sample collection is directly observed by CM programme staff. The voucher for the initial sample that was non‐reactive for methamphetamine and cocaine metabolites was worth $2.00. Vouchers increased in value by 25 cents for each consecutive stimulant‐free sample to a maximum of $10.00. Participants earned an $8.50 bonus voucher for every third consecutive stimulant‐free sample. Participants who provided a reactive urine toxicology result for stimulants could return to their place in the escalating reinforcement schedule after producing three consecutive urine samples that were non‐reactive for methamphetamine and cocaine metabolites. The total possible reinforcement of stimulant abstinence during thrice‐weekly urine screening over the three months was $330.

### 2.5 Positive affect intervention

Affect Regulation Treatment to Enhance Methamphetamine Intervention Success (ARTEMIS) is a multi‐component, individually delivered intervention that consists of five 1‐hour sessions delivered during the 3‐month CM period. Table 1 provides a brief overview of the intervention protocol, and more detailed information is provided elsewhere 32. The ARTEMIS intervention protocol consists of eight core skills that have been shown to increase positive affect and improve psychological adjustment in prior clinical research 24. Informed by prior research examining the efficacy of mindfulness‐based relapse prevention 30, participants also completed meditation exercises during ARTEMIS intervention sessions to further enhance mindfulness and assist individuals in coping more effectively with methamphetamine withdrawal. Participants received a workbook and an iPod shuffle that was pre‐loaded with meditation exercises. Participants received $20 cash for completing each individual session, and 49 (89%) completed all five sessions.

**Table 1:** Positive affect intervention protocol for sexual minority men living with HIV who use methamphetamine

Session	Positive affect skills	Additional intervention content
1	Noticing positive events Capitalizing on positive events Gratitude	Psychoeducation on stimulant withdrawal Capitalizing on non‐reactive urine toxicology Breathing retraining with positive event imagery
2	Mindfulness (informal and formal)	Self‐compassion Breath meditation
3	Positive reappraisal	Problem‐focused coping & reasoned action Breath meditation
4	Strengths Attainable goals	Values clarification Mountain meditation
5	Altruism	Pursuing volunteer opportunities Linkage to community‐based services Loving kindness meditation

### 2.6 Attention‐control condition

The attention‐control condition consisted of five sessions that included face‐to‐face administration of psychological measures and neutral writing exercises 34. We chose an attention‐control to provide comparable contact time with study staff and identical incentives. Participants were instructed to write as if they were reporting facts without going into thoughts or feelings about the events (e.g. plans for the next 24 hours). Participants received $20 cash for completing each attention‐control session and an iPod with three pre‐loaded popular songs. A total of 49 (89%) participants completed all five attention‐control sessions.

### 2.7 Fidelity monitoring

Facilitators with master's level training in public health or counselling were provided with a detailed manual and completed a 1‐day training with ongoing mock sessions to achieve competency in the delivery of the intervention. All ARTEMIS intervention sessions were audio recorded. Audio recordings of ARTEMIS intervention sessions were reviewed by a clinical supervisor during weekly individual supervision to provide feedback on delivery of intervention content and process‐oriented techniques. Monthly group supervision meetings provided opportunities for case presentation and ongoing discussions about optimizing the delivery of both the ARTEMIS intervention skills and attention‐control protocol. Audio recordings of intervention sessions were reviewed by an independent fidelity monitor to provide more detailed feedback to facilitators regarding adherence to the ARTEMIS intervention content, interpersonal skills, rapport and session flow. A total of 71 of the 259 completed ARTEMIS intervention sessions (27%) were coded using fidelity rating checklists with detailed feedback provided to facilitators.

### 2.8 HIV disease markers

The primary outcome was log_10_ HIV viral load, which was measured using the Abbott RealTime HIV‐1 reverse transcription‐polymerase chain reaction assay. This assay reliably detects HIV RNA from 40 to 10,000,000 copies/mL. We also measured whether participants displayed any unsuppressed HIV viral load (≥200 copies/mL) because this is the level at which there is increased odds of onward HIV transmission during condomless sex 2. CD4^+^ T‐cell count was measured by Quest Diagnostics using flow cytometry.

### 2.9 Positive affect

The Differential Emotions Scale, which was modified to include additional positive affect items 22, 35. Participants rated how frequently they felt a particular affect in the past week from zero (never) to four (most of the time). The positive affect measure included 14 items (Cronbach's alpha = 0.89).

### 2.10 Stimulant use and methamphetamine craving

Participants reported how often they used methamphetamine, powder cocaine and crack‐cocaine in the past three months. Each stimulant was rated separately on a Likert‐type scale from zero (not at all) to seven (daily). Where participants reported using multiple stimulants, the highest frequency rating was selected for the composite outcome. Urine samples provided by participants were also collected for on site toxicology screening of methamphetamine and cocaine metabolites using the iCup (Redwood Biotech, Inc.; Santa Rosa, CA). Reactive urine toxicology results are indicative of stimulant use within the past 72 hours. Finally, the Penn Alcohol Craving Scale was adapted for assessment of methamphetamine craving 36. Frequency, intensity and duration of thoughts about using methamphetamine were assessed (Cronbach's alpha = 0.90).

### 2.11 Statistical analyses

We utilized the non‐parametric Wilcoxon test of means and chi‐squares (Fisher's exact chi‐square where cell counts were less than five) to determine whether the experimental conditions were balanced at baseline. Stata version 15 was used to perform the analyses. Statistical significance was set at *p* < 0.05 (two‐tailed). Intent‐to‐treat analyses for continuous outcome variables compared the experimental conditions across time by testing the group‐by‐time interaction effects using repeated measures models with correlated residuals estimated via maximum likelihood using the Stata‐mixed‐command. Cases with partial data were included under the missing at random assumption via direct maximum likelihood estimation. The correlation structure for residuals was selected by comparing the Bayesian information criterion (BIC) statistics among available correlation structures in Stata suitable for repeated measures data with unequally‐spaced time points – with and without the assumption of equivalent correlation structures across the two treatment groups: independent, exchangeable, exponential, and unstructured. The correlation structure with the lowest BIC statistic was selected for testing the equality of means across treatment groups and time. For the reactive urine toxicology result for stimulants binary outcome, generalized linear mixed models (GLMMs) were fitted; a GLMM with random intercepts and slopes was compared with a GLMM with random intercepts only using the BIC statistic. Effects included in each model were intervention condition, time, and their interaction. To avoid assuming linearity of effects over time, both group and time effects were treated as categorical variables. Planned simple main effects tests compared the ARTEMIS intervention and attention‐control conditions at each follow‐up assessment using the Stata‐contrast‐post‐estimation command. To investigate the potential decay of intervention effects over time, we performed a sensitivity analysis for positive affect that repeated the original analysis with the 15‐month assessment excluded. Finally, to estimate the relative risk of one or more instances of unsuppressed viral load as well as one or more instances of a reactive urine toxicology result for stimulants across the 15‐month investigation period, logistic regression after baseline onto intervention condition was performed via the Stata‐logistic‐command followed by the Stata user‐written‐adjrr‐post‐estimation command to obtain the risk ratio (RR).

Using NCSS PASS with a total sample size of 150, 80% retention, four repeated measures of viral load, and at varying levels of autocorrelation, the minimum detectable effect sizes are in the small‐medium range (Cohen's *d* = 0.29 to 0.47). Overall, this RCT had adequate power to detect moderate effects of the ARTEMIS intervention on the primary outcome over the 15‐month follow‐up.

## 3 Results

Among the 110 randomized participants, age ranged from 24 to 59 years with a mean of 43.2 (SD = 8.9). Close to half of participants were Caucasian (43%), 29% were Hispanic/Latino, 16% were African American and 12% were other ethnic minorities or multiracial. The majority of participants completed at least some college (75%) and 65% had an income of less than $16,000 USD per year. The median baseline CD4^+^ T‐cell count was 646 (Interquartile Range = 428 to 816) cells/mm^3^ and 14% had an unsuppressed viral load (≥200 copies/mL) at baseline. Participants had been living with HIV for an average of 12.9 (SD = 8.6) years and most were currently prescribed ART at baseline (89%). Table 2 summarizes demographics as well as health status indicators at baseline for the ARTEMIS intervention and attention‐control conditions separately.

**Table 2:** Baseline characteristics of trial participants (N = 110)

	ARTEMIS (n = 55)	Attention‐control (n = 55)	*p*‐Value
	M (SD)	M (SD)	
Age	43.2 (9.2)	43.2 (8.5)	0.88
Time since HIV diagnosis (years)	13.4 (8.9)	12.5 (8.4)	0.62
CD4^+^ T‐cell count (cells/mm^3^)	642.9 (272.8)	639.9 (313.6)	0.99

	n (%)	n (%)	
Race/ethnicity			
Black/African American	9 (8.2)	9 (8.2)	0.12
White	18 (32.7)	29 (52.7)	
Hispanic/Latino	21 (38.2)	11 (20.0)	
Other ethnic minority	7 (12.7)	6 (10.9)	
Education			
Less than high school	5 (9.1)	3 (5.5)	0.62
HS graduate	8 (14.6)	9 (16.4)	
Some college/trade school	25 (45.5)	32 (58.2)	
College graduate	10 (18.2)	7 (12.7)	
Post graduate	7 (12.7)	4 (7.3)	
Income			
<$4999	8 (14.8)	8 (14.6)	0.26
$5,000 to $11,999	9 (16.7)	19 (34.6)	
$12,000 to $15,999	14 (25.9)	13 (23.6)	
$16,000 to $24,999	6 (11.1)	6 (10.9)	
$25,000 to $34,999	4 (7.4)	5 (9.1)	
$35,000 to $49,999	7 (13.0)	2 (3.6)	
>$50,000	6 (11.1)	2 (3.6)	
Prescribed anti‐retroviral therapy	49 (89.1)	49 (89.1)	1.00
HIV viral load ≥200 copies/mL	6 (11.1)	9 (16.7)	0.14

ARTEMIS, affect regulation intervention to enhance methamphetamine intervention success.

**Figure 2:**
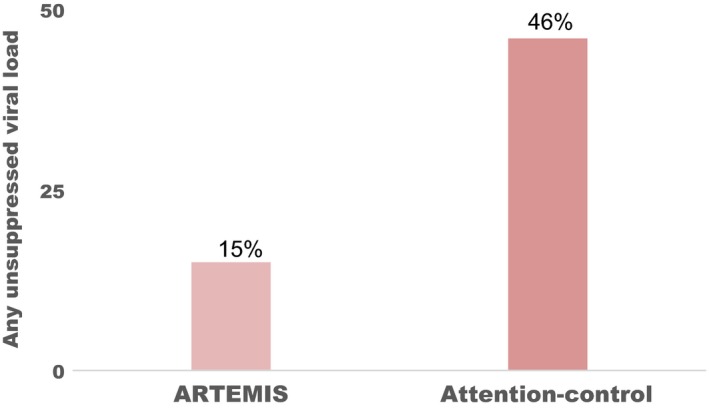
Any unsuppressed HIV viral load (≥200 copies/mL) over 15 months by treatment arm.

As shown in Table 3, we observed a significant group‐by‐time interaction for the primary outcome of log_10_ viral load (χ^2^(3) = 7.83, *p* = 0.049). Planned comparisons demonstrated that the ARTEMIS intervention participants displayed significantly lower log_10_ viral load at 6 (z = 4.11; *p* < 0.001), 12 (z = 2.60; *p* = 0.009), and 15 months (z = 2.41; *p* = 0.016). These *p*‐values for log_10_ viral load remained significant (*p* < 0.05) even after adjusting for multiple testing. As shown in Figure 2, we also observed via logistic regression that ARTEMIS intervention participants had significantly lower risk of one or more unsuppressed viral load measurements over the 15‐month follow‐up period (RR = 0.33; 95% CI = 0.15 to 0.69; *p* < 0.001). There were no significant differences in CD4^+^ T‐cell count by treatment arm (χ^2^(3) = 1.95, *p* = 0.584), but there was a marginally significant group‐by‐time interaction for positive affect (χ^2^(8) = 14.03, *p* = 0.051). A sensitivity analysis that excluded the 15‐month time point identified a significant group‐by‐time interaction for positive affect (χ^2^(6) = 12.96, *p* = 0.044) with significantly higher positive affect among ARTEMIS intervention participants at session 5 (z = 2.63; *p* = 0.009) as well as at 6 (z = 2.67; *p* = 0.008) and 12 (z = 2.10; *p* = 0.036) months.

**Table 3:** Changes in HIV disease markers and positive affect by treatment arm (N = 110)

	N	ARTEMIS (n = 55)	Attention‐control (N = 55)	Cohen's *d* (95% CI)	Group × time *p*‐Value
		M (SD)	M (SD)		
HIV viral load (Log_10_)					
Baseline	108	1.04 (1.23)	1.42 (1.40)	–	0.049
6 Months	87	0.69 (0.75)^a^	1.82 (1.61)^a^	0.89 (0.45, 1.33)	
12 Months	84	0.93 (1.21)^b^	1.52 (1.51)^b^	0.43 (−0.01, 0.86)	
15 Months	74	0.88 (1.03)^b^	1.49 (1.38)^b^	0.50 (0.04, 0.96)	
CD4^+^ T−cell count (square root)					
Baseline	108	24.68 (5.86)	24.18 (7.50)	–	0.584
6 Months	85	25.28 (6.08)	23.06 (7.83)	0.32 (−0.11, 0.74)	
12 Months	78	24.10 (6.41)	23.93 (8.31)	0.02 (−0.42, 0.47)	
15 Months	70	25.13 (5.61)	23.80 (7.29)	0.20 (−0.27, 0.67)	
Positive affect					
Screening	110	33.27 (7.63)	31.25 (8.76)	–	0.051
Baseline	110	32.38 (8.64)	31.40 (9.41)	–	
Session 1	110	30.24 (8.97)	30.02 (10.11)	0.02 (−0.35, 0.40)	
Session 3	104	33.33 (8.00)	29.75 (9.85)	0.40 (0.01, 0.79)	
Session 5	98	35.06 (9.23)^a^	29.57 (10.34)^a^	0.56 (0.16, 0.96)	
3 Months	98	34.82 (8.42)	32.27 (9.47)	0.28 (−0.11, 0.68)	
6 Months	96	35.45 (8.12)^a^	30.96 (8.84)^a^	0.53 (0.12, 0.93)	
12 Months	88	35.17 (10.11)^b^	30.74 (11.19)^b^	0.41 (−0.01, 0.84)	
15 Months	78	32.86 (10.11)	32.26 (12.07)	0.05 (−0.39, 0.50)	

ARTEMIS, affect regulation intervention to enhance methamphetamine intervention success; between group differences within each time point:

a *p* < 0.01;

b *p* ≤ 0.05. *p*‐values were generated from a repeated measures analysis with correlated residuals fitted with the mixed‐command in Stata 15. The best fitting correlation structure for each outcome was exponential with separate variances and correlations by treatment group for HIV viral load and exchangeable for CD4^+^ T−cell count and positive affect.

As shown in Table 4, we observed a significant group‐by‐time interaction for self‐reported frequency of stimulant use (χ^2^(5) = 12.28, *p* = 0.031) and reactive urine toxicology results for stimulants (χ^2^(5) = 13.75, *p* = 0.017). ARTEMIS intervention participants reported decreases in the frequency of stimulant use at 3 (z = −2.17; *p* = 0.030), 6 (z = −2.21; *p* = 0.027) and 12 (z = −2.11; *p* = 0.035) months. There were no significant differences in the proportion of ARTEMIS intervention versus attention‐control participants providing a reactive urine sample for stimulants at each follow‐up assessment. Although ARTEMIS intervention participants had lower risk of providing one or more urine samples that were reactive for stimulants over the 15‐month follow‐up period, this did not reach statistical significance (RR = 0.80; 95% CI = 0.63 to 1.02; *p* = 0.056). The group‐by‐time interaction for methamphetamine craving did not reach statistical significance (χ^2^(4) = 7.77, *p* = 0.100).

**Table 4:** Changes in stimulant use and methamphetamine craving by treatment arm (N = 110)

	N	ARTEMIS (n = 55)	Attention‐control (n = 55)	Cohen's *d* (95% CI)	Group × time *p*‐value
		M (SD)	M (SD)		
Self‐reported stimulant use (past three months)					
Screening	110	4.65 (1.65)	4.51 (1.87)	–	0.031
Baseline	110	4.16 (1.89)	4.09 (1.94)	–	
3 Months	98	2.22 (2.16)^a^	3.23 (2.26)^a^	0.46 (0.05, 0.86)	
6 Months	96	2.40 (2.19)^a^	3.39 (2.23)^a^	0.44 (0.04, 0.85)	
12 Months	88	2.48 (2.23)^a^	3.39 (2.43)^a^	0.39 (−0.03, 0.81)	
15 Months	78	2.65 (2.38)	3.10 (2.45)	0.18 (−0.27, 0.63)	
Methamphetamine craving					
Baseline	110	2.62 (1.31)	2.85 (1.56)	–	0.100
3 Months	98	1.79 (1.36)	2.56 (1.69)	0.50 (0.10, 0.90)	
6 Months	96	1.77 (1.31)	2.68 (1.92)	0.55 (0.14, 0.96)	
12 Months	88	1.59 (1.37)	2.61 (1.88)	0.61 (0.18, 1.04)	
15 Months	78	2.18 (1.74)	2.39 (1.77)	0.12 (−0.32, 0.57)	

		N (%)	N (%)	Cohen's *h* (95% CI)	
Reactive urine toxicology for stimulants					
Screening	110	33 (60)	23 (42)	–	0.017
Baseline	110	26 (47)	27 (49)	–	
3 Months	98	24 (49)	23 (54)	0.09 (−0.32, 0.50)	
6 Months	96	22 (50)	29 (66)	0.32 (−0.09, 0.74)	
12 Months	88	17 (45)	28 (64)	0.38 (−0.05, 0.82)	
15 Months	78	19 (54)	23 (56)	0.04 (−0.41, 0.49)	

ARTEMIS, affect regulation intervention to enhance methamphetamine intervention success; Tox+ = reactive for stimulant metabolites; between group differences within each time point:

a *p* < 0.05. *p*‐values were generated from a repeated measures analysis with correlated residuals fitted with the mixed command in Stata 15. The best fitting correlation structures for continuous outcomes were exchangeable for stimulant use in the past three months and unstructured with separate variances and correlations by treatment group for Change in Stimulant Craving. The random intercepts only model performed better than the more complex random intercepts and slopes model for the binary Urine Tox + for Stimulants outcome.

## 4 Discussion

This RCT is the first to demonstrate the durable efficacy of a behavioural intervention for optimizing the clinical and public health benefits of TasP with people who use substances. Although prior RCTs support the short‐term effectiveness of CM interventions for decreasing viral load in people who use substances 15, 16, these effects were not maintained after tangible incentives for behaviour change were discontinued. Results of the present RCT conducted with sexual minority men living with HIV who use methamphetamine demonstrated that delivering a 5‐session ARTEMIS intervention during three months of community‐based CM for stimulant abstinence achieved durable reductions in viral load and lower risk of any unsuppressed viral load over 15 months. Interestingly, we observed intervention‐related reductions in viral load despite the fact that only 14% of participants presented with unsuppressed viral load at baseline. This highlights there may be important clinical benefits of integrative, behavioural interventions to mitigate risk of viral rebound in people living with HIV who use substances that are initially virally suppressed.

The efficacy of delivering the ARTEMIS intervention during community‐based CM is further supported by intent‐to‐treat analyses of secondary outcomes. Those randomized to receive the ARTEMIS intervention during CM reported significant increases in positive affect as well as decreases in stimulant use at six and twelve months. The potential promise of this approach is further supported by a recent pilot RCT with HIV‐negative sexual minority men who use methamphetamine where a behavioural activation intervention achieved short‐term reductions in self‐reported methamphetamine use and condomless anal sex 37. Further clinical research is necessary to test the efficacy of low intensity methods such as mHealth applications for achieving more durable increases in positive affect and reductions in stimulant use. Future studies should also examine whether intervention‐related changes in positive affect, stimulant use, and other secondary outcomes mediate the durable efficacy of the ARTEMIS intervention on viral suppression.

The scientific rigor of this RCT is consistent with other high quality RCTs of behavioural and biomedical interventions that enrolled people living with HIV who use substances 15, 16, 38. We achieved strong rates of engagement in the ARTEMIS intervention, attention‐control condition, and CM as well as long‐term follow‐up rates comparable to a recently published multi‐site RCT with a diverse sample of people living with HIV who use substances 15. In contrast to other RCTs of behavioural interventions with this population 39, we also required biological confirmation of recent methamphetamine use for enrolment. This maximizes internal validity by minimizing the likelihood that participants are merely reporting methamphetamine use to receive CM and research incentives. We also implemented a run‐in period to ensure that all randomized participants were sufficiently engaged in the RCT, which did not meaningfully diminish our rate of randomization. Finally, our team tested the efficacy of the positive affect intervention in partnership with a community‐based CM programme, which supports the potential for broader implementation.

Findings from this efficacy RCT should also be interpreted in context of some limitations. Consistent with prior single site RCTs with this population 40, the sample size was modest and only sexual minority men living with HIV who use methamphetamine were enrolled. It is also noteworthy that this RCT was conducted in San Francisco, a well‐resourced setting with extensive services for sexual minority men living with HIV who use methamphetamine 41. This may partially explain the low prevalence of unsuppressed viral load observed at baseline. Although approximately half of participants were ethnic minority men, further research is necessary to test culturally tailored approaches to optimize the benefits of TasP in sexual minority men who use stimulants in domestic as well as international settings. There is a clear need for a Phase III RCT to examine the effectiveness of this integrative, behavioural intervention for achieving durable viral suppression in a more generalizable sample. Subsequent RCTs should also focus on testing the effectiveness of this integrative, behavioural intervention in the broader population of people living with HIV who use stimulants and employ experimental procedures that more closely resemble treatment settings (i.e. no run‐in period, no incentives for attending individual sessions). Examination of the effects of the intervention on objective metrics of ART adherence (e.g. via hair or urine metrics) would strengthen the scientific rigor of future RCTs 42, 43. Further clinical research is also needed to examine the potential benefits of augmenting this integrative, behavioural intervention with promising medications for methamphetamine users such as mirtazapine 40.

## 5 Conclusions

This RCT provides compelling support for the efficacy of an integrative, behavioural intervention for achieving sustained and clinically meaningful reductions in viral load among sexual minority men living with HIV who use methamphetamine. In the era of TasP, comprehensive approaches to achieve and maintain viral suppression in this high priority population have important implications for improving health outcomes and substantially reducing risk of onward HIV transmission. Findings will inform future RCTs testing the effectiveness of integrative, behavioural interventions to optimize HIV/AIDS treatment and prevention among people living with HIV who use substances.

## Competing interest

The authors have no conflicts of interest to report. This project was investigator initiated without directives from the funding sources in design and conduct of the study; collection, management, analysis and interpretation of the data; preparation, review, or approval of the manuscript; and decision to submit the manuscript for publication.

## Authors' contributions

AWC, JTM and WJW developed and refined hypotheses for this project. TBN, SED and JLE led data management and statistical analyses for this project with feedback on analytic plan provided by DJF. WG and JJ assisted with implementing and refining the protocol for this trial. SS provided feedback throughout the randomized controlled trial regarding assessments, contingency management methods, and interpretation of findings. LC served as a project director during the latter stages of this trial and led efforts to obtain viral load information from the medical record. MG providing protocols and support necessary to collect hair specimens to verify recent methamphetamine use in this trial. KJH contributed expertise in adherence intervention research with this population and provided feedback relevant to interpretation of findings. MVD and RA led community‐based CM at the San Francisco AIDS Foundation as well as assisted with refining the ARTEMIS positive affect intervention protocol. AWC led this manuscript. All authors provided feedback on the manuscript and approved of the final manuscript before submission.
